# The impact of task complexity and translating self-efficacy belief on students’ translation performance: Evidence from process and product data

**DOI:** 10.3389/fpsyg.2022.911850

**Published:** 2022-11-03

**Authors:** Xiangyan Zhou, Xiangling Wang, Xiaodong Liu

**Affiliations:** ^1^College of Foreign Languages, Hunan University, Changsha, China; ^2^School of Foreign Studies, Hunan University of Humanities, Science and Technology, Loudi, China

**Keywords:** task complexity, translating self-efficacy belief, interaction, translation process, product quality

## Abstract

Previous studies that explored the impact of task-related variables on translation performance focused on task complexity but reported inconsistent findings. This study shows that, to understand the effect of task complexity on translation process and its end product, performance in translation tasks of various complexity levels needs to be compared in a specific setting, in which more factors are considered besides task complexity—especially students’ translating self-efficacy belief (TSEB). Data obtained from screen recording, subjective rating, semi-structured interview, and quality evaluation were triangulated to measure how task complexity influenced the translation performance of Chinese students with high and low TSEB. We found that the complex task led to significantly longer task duration, greater self-reported cognitive effort, lower accuracy, and poorer fluency than the simple one among students, irrespective of their TSEB level. Besides, the high-TSEB group outperformed the low-TSEB group in translation accuracy and fluency in both tasks. However, the interaction effect of task complexity and TSEB was not significant, due possibly to weak problem awareness among students. Our study has implications for effectively designing task complexity, getting the benefits of TSEB, and improving research on translation performance.

## Introduction

Translation process research, which has been ongoing for about 40 years, focuses on human cognition during translation (Alves and Hurtado Albir, 2010; Jääskeläinen, 2016). In translation process research, simultaneous analysis of the translation process and its end product is vitally important since “looking only at the process or the product…is looking at only one side of a coin” (O’Brien, 2013: 6). Translators, related both to the process and product of translation, have thus attracted great attention in translation process research (e.g., Muñoz Martín, 2014; Lehka-Paul and Whyatt, 2016; Araghian et al., 2018). Although several factors can affect translators’ mental process and product quality, previous studies have identified the task itself as a key factor (e.g., Kelly, 2005; O’Brien, 2013). First, there is a need for translation teachers to design appropriate tasks as per pedagogical objectives. However, traditional translator training is often criticized for the lack of appropriate criteria for source text selection and task design in general (Kelly, 2005). In addition, task design is also an important element in research design (O’Brien, 2013) as tasks adopted shall be appropriate for the research project. The past decades have seen a growing interest in investigating how task properties may influence translators’ performance. Researchers have explored task type (e.g., Jia et al., 2019), task modality (e.g., Chmiel et al., 2020), task condition (e.g., Weng et al., 2022), and task complexity (e.g., Feng, 2017; Sun et al., 2020). Among them, studies dealing with task complexity have remained a major focus and reported interesting, albeit inconsistent, findings.

Although a major line of task-based research investigated the impact of task complexity, it focused primarily on the translation process and largely did not relate its findings to specific translator factors (e.g., Feng, 2017; Liu et al., 2019). Translation has often been depicted as a cognitive task driven by problem-solving (Angelone, 2018). As translation problems are individual and arise from the interplay between task properties and characteristics of task performers (Muñoz Martín and Olalla-Soler, 2022), translators may deliver varied performance even on the same task. Studies that investigated the interplay between task properties and translator characteristics worked on such individual differences as L2 proficiency (Pokorn et al., 2019), working memory capacity (Wang, 2022), and emotional intelligence (Ghobadi et al., 2021), among others. However, to the best of our knowledge, no study has so far investigated how task complexity influences the translation performance of students with different levels of self-efficacy belief, an important affective variable influencing students’ motivation and learning (Bandura, 1997) and a construct recently introduced into translation studies (Yang et al., 2021a). This study attempts to contribute further empirical data to translation performance research by analyzing both the process and the product of written translation and by considering the interaction of task complexity and translating self-efficacy belief (TSEB).

## Literature review

### Tapping into the process and product data of translation performance

Literature regarding the nature of translation performance reveals two assessment approaches: product-and behavior-based assessment. Most studies perceived translation performance as the result of translation activities and assessed it by evaluating the quality of the products only (Jääskeläinen, 2016). While exploring the relationship between personality type and performance in translating expressive, appellative and informative texts, Shaki and Khoshsaligheh (2017) concluded that the sensing-type students delivered less successful performance than the intuitive-type students in all three tasks. Their performance was evaluated against Waddington’s (2001) rubric, which was also adopted by Ghobadi et al. (2021) to assess participants’ translation performance. Meanwhile, some studies considered translation performance as the sum of behaviors that participants controlled in the process of translation (Muñoz Martín, 2014). Therefore, participants’ translation performance was understood by evaluating their behaviors in the translation process. For example, to better understand the mental processes involved in translation, Lörscher (1991) investigated the strategic translation performance of 56 secondary school and university students by utilizing think-aloud protocols. Besides, Rothe-Neves (2003) analyzed the process features of translation performance with processing time and writing effort measures.

To solve the riddles of translation as a process and a product, there is a need to combine quality assessment and process findings for performance evaluation. This is in line with the suggestion of Jääskeläinen (2016) and Angelone (2018) that translation performance research should be grounded in both process and product data. Combining the two data sources opens up new research avenues and increases the possibilities of finding explanations and generalizing results to real-life circumstances. It may help explain, for instance, why longer task duration sometimes leads to better performance, but sometimes to poorer performance among the same group of students (e.g., Seufert et al., 2017). Despite potential benefits of using the integrated approach, product quality assessment has been integrated with only a few process-oriented studies (Saldanha and O’Brien, 2014). This study aims to demonstrate the utility of this approach by collecting empirical evidence on both the translation process and its end product.

### Task complexity and translation performance

Task complexity, defined as “the result of the attentional, memory, reasoning, and other information-processing demands imposed by the structure of the task” on task performers (Robinson, 2011: 106), can affect cognitive processing (Plass et al., 2010). Translation is a high-order cognitive task that imposes cognitive load on and engages cognitive effort of task performers (Liu et al., 2019). Therefore, we must first differentiate cognitive load and cognitive effort before discussing the relationship between task complexity and translation performance. In the present study, cognitive load is associated with the complexity of a task as it refers to the demand for cognitive resources imposed on students by the task, and cognitive effort associated with the actual response by a student as it is the amount of cognitive resources that the student expends to accomplish the task. This is consistent with the constructs of cognitive load and cognitive effort developed in educational psychology (Sweller et al., 1998). While the cognitive load of a task can theoretically be identical for different students (Liu et al., 2019; Ehrensberger-Dow et al., 2020), the cognitive effort expended in a task is individual since students have certain freedom regarding how much effort to expend and how to expend it (Feng, 2017; Sun et al., 2020).

Previous research shows that the highest level of cognitive effort and task performance occur when the task imposes moderate cognitive load (e.g., Plass et al., 2010; Chen et al., 2016). Therefore, translation tasks can optimize students’ opportunities for performance and development if they are of moderate complexity. Such a claim aligns with the social constructivist approach to translator education, where Kiraly emphasizes the use of scaffolded learning activities (Kiraly, 2000). By far, task complexity has been an issue central to curriculum and test development in translator education (Sun and Shreve, 2014). Myriad factors contribute to the complexity of translation tasks, such as source text complexity (Sun and Shreve, 2014), source text quality (Ehrensberger-Dow et al., 2020), the number of simultaneous tasks (Sun et al., 2020), task familiarity (Pokorn et al., 2019), and directionality (Whyatt, 2019). Among them, source text complexity has been a constant focus. To investigate the level of text complexity, researchers have resorted to various measures, including readability (Sun and Shreve, 2014; Whyatt, 2019), word frequency and non-literalness (Liu et al., 2019), degree of polysemy (Mishra et al., 2013), dependency distance (Liang et al., 2017), text structure (Yuan, 2022), and cohesion (Wu, 2019).

Although numerous studies have analyzed how task complexity affects translation performance, the research findings are conflicting. For example, by operationalizing task complexity as the number of simultaneous tasks, Sun et al. (2020) concluded that compared with translating silently, translating while thinking aloud resulted in a higher level of cognitive effort as indicated by task duration, fixation duration and self-ratings; however, the dual-task condition had no influence on translation quality when the source text was complex. Sun et al.’s (2020) findings were only partly consistent with the findings of Wu (2019). In Wu’s (2019) study, task complexity, operationalized as several text characteristics, was positively correlated with self-reported cognitive effort but negatively correlated with translation quality. Despite their discrepancy, Sun et al. (2020) and Wu (2019) revealed that students devoted a higher level of cognitive effort with an increase in task complexity. When task complexity was operationalized as directionality, according to Fonseca (2015), the consensus among translators and translation scholars was that translating into a non-native language (also known as L2 translation) was cognitively more demanding than translating into the native language (also known as L1 translation). However, empirical studies dealing with the effect of directionality on translation performance also reported inconsistent findings (Whyatt, 2019). One possible reason underlying such inconsistency is that existing research on the impact of task complexity does not consider its interplay with translator characteristics.

### Self-efficacy belief in translation

How task performance is influenced by the interaction between task properties and learner characteristics has been consistently studied in second language acquisition (Robinson, 2011) and educational psychology (Sweller et al., 1998). Given that individual differences may influence how many cognitive resources to devote and how to expend them in task implementation (e.g., Hoffman and Schraw, 2009; Wang, 2022), there is a need to study translation performance by giving due consideration to translator characteristics. Among the various translator factors, it is critical to examine whether self-efficacy interacts with task complexity in influencing task performance—an observation made in prior research in other disciplines (e.g., Judge et al., 2007; Hoffman and Schraw, 2009; Rahimi and Zhang, 2019). Interestingly, while Judge et al. (2007) reported that the benefits of self-efficacy were difficult to realize in more complex tasks, some studies concluded that the role of self-efficacy was more manifest when task complexity was higher (e.g., Hoffman and Schraw, 2009; Rahimi and Zhang, 2019).

As an affective factor influencing cognitive and motivational processes (Bandura, 1997), self-efficacy can motivate learners and encourage them to put in more effort once an action has been initiated (Bandura, 1995). However, the construct has only recently begun to draw attention from researchers in the field of translation (e.g., Bolaños-Medina, 2014; Muñoz Martín, 2014; Bolaños-Medina and Núñez, 2018). Muñoz Martín (2014) attached importance to the correlation between self-efficacy and translation expertise by specifically including self-efficacy as one of the minimal sub-dimensions of self-concept, which constitutes translation expertise together with knowledge, adaptive psychophysiological traits, problem-solving skills, and regulatory skills. Bolaños-Medina (2014) also proposed that self-efficacy was a construct of relevance for translation process research, related particularly to proficient source language reading comprehension, tolerance of ambiguity, general text translation, and documentation abilities. Besides, Moores et al. (2006) pointed out that an understanding of self-efficacy was required if training programs were designed to develop expert performance level in complex tasks.

Notably, the one-measure-fit-all approach usually has constrained explanatory and predictive value because “most of the items in an all-purpose test may have little or no relevance to the domain of functioning” (Bandura, 2006: 307). This idea aligns with Alves and Hurtado Albir’s (2010: 34) proposal that translation process research should “design its own instruments for data collection.” Therefore, empirical research on self-efficacy belief in translation shall utilize measurement scales tailored for translation tasks. So far, several such scales have been developed (e.g., Bolaños-Medina and Núñez, 2018; Yang et al., 2021a). Since this study focused on the Chinese-English language pair, the Translating Self-Efficacy China scale developed by Yang et al. (2021a) was adopted, which was specifically designed for students with Chinese as their mother tongue and English as a foreign language.

### The present study

**Figure 1:**
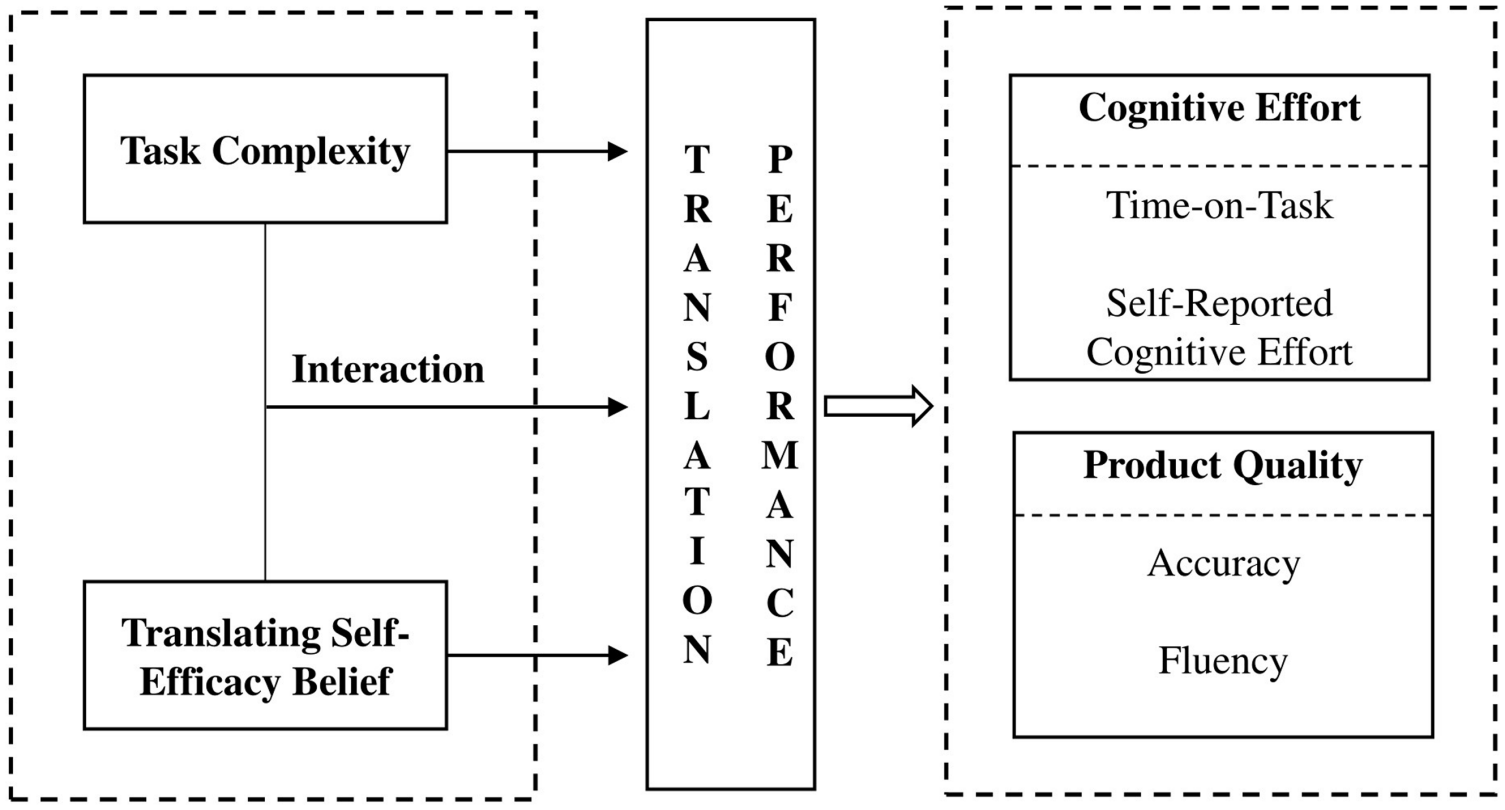
Proposed research framework of translation performance.

Considering the limitations in earlier studies, the current study attempts to provide further empirical evidence on translation performance. It collects data on both the process and product of two written translation tasks, which are of different complexity levels and performed by homogeneous groups of students with high and low TSEB. A framework is proposed to delineate variables contributing to and measures of translation performance in the current study (see Figure 1). We promote the idea that task complexity and TSEB influence both students’ mental process and product quality; moreover, task complexity might interact with TSEB in influencing their translation performance. For process features of translation performance, we adopt two measures of cognitive effort following previous research (Ehrensberger-Dow et al., 2020; Sun et al., 2020): time-on-task and self-reported cognitive effort. Regarding the quality of translation products, accuracy and fluency are discussed against assessment guidelines.

In brief, to investigate how task complexity and TSEB influence students’ cognitive effort and product quality, and whether there is an interaction effect between the two independent variables, the following research questions (RQs) are raised:

**RQ1:** What is the impact of task complexity on cognitive effort and product quality of students?

**RQ2:** How does TSEB influence students’ cognitive effort and product quality?

**RQ3:** Does task complexity interact with TSEB in influencing students’ translation performance? If yes, how?

## Materials and methods

### Participants

Brysbaert (2019) proposed that an experiment involving interaction required a minimum sample size of 100 in psychology research. Thus, 136 second-year translation students were recruited for the study on a voluntary basis. The students were from one comprehensive university in mainland China. They all enrolled in the “Translation Theory and Practice” course, which constituted their first experience of intensive translation training after a foundation year with modules in their two working languages (Chinese as their mother tongue and English as a foreign language), an introduction to linguistics for translation, and instrumental skills such as documentary research and computer skills. When the experiment was conducted, the participants had learned English for about 10 years. Thus, they were generally equipped with basic translation skills and language abilities that could guarantee their completion of the translation tasks. The students were aged between 19 and 22, and their gender was primarily female (*N* = 116, 85.3%).

Based on the measures of TSEB, students were divided into two groups as per the guideline of median split (Malik et al., 2021). Namely, the top 50% students (*N* = 68) with their TSEB value above the median value were assigned to the high-TSEB group, while the remaining 50% (*N* = 68) assigned to the low-TSEB group. Participants’ CET4 score, a national test designed to measure English proficiency of undergraduates in China, was used as a measure of their L2 proficiency. The results of the independent samples *t*-test showed that the two groups were significantly different in TSEB (*t* = −13.704, *p* < 0.001) and in L2 proficiency (*t* = −2.297, *p* < 0.05).

Students were involved for pedagogical considerations. The research outcome is expected to help translation teachers make informed decisions in translation task selection and help students benefit from high TSEB for performance improvement. Task selection is crucial for translator training, particularly at the initial training stage, as unrealistically complex tasks prove to be a source of frustration for students (Kelly, 2005; Wu, 2019).

### Translation tasks

#### Translation direction

Demand for translation from Chinese into English has remained strong in China. According to Kelly (2000), translation into a non-mother tongue is a professional necessity in many local translation markets and a useful training exercise that contributes to students’ understanding of translation problems. However, despite the presence of L2 translation on the market, the performance of L2 translation remains under-researched (Pokorn et al., 2019). In the hope of contributing to research on L2 translation, we decided to implement Chinese-to-English as the translation direction.

#### Text selection

The current study operationalized task complexity as quantifiable measures of text characteristics following Wu’s (2019) suggestion. The two source texts are both about mobile phones and between 160 and 170 Chinese characters (see Appendix A for details). This precludes the possibility that unfamiliarity with the subject domain would skew task performance. The selection of quantifiable measures was guided by literature review. First, lexical polysemy indicates translation ambiguity and task complexity (Mishra et al., 2013). As a word with more senses may be ambiguous and thus slow down processing for learners with a low level of skill and knowledge (McNamara et al., 2014), word polysemy value is positively correlated with task processing demands. Second, low cohesion may increase reading time and disrupt comprehension (McNamara et al., 2014). Connectives are very important in establishing cohesion (Graesser et al., 2011). In Chinese-to-English interpreting, connectives were added to enhance cohesion by professional translators, so as to make implicit information in the Chinese text explicit in the English text (Tang and Li, 2017). Therefore, the incidence score of connectives in the Chinese text is reversely linked to task processing demands.

We utilized the Coh-Metrix Web Tool (Traditional Chinese version) to analyze properties of the two source texts. Coh-Metrix, a linguistic workbench that uses indices to scale texts on characteristics related to words, sentences, and connections between sentences, has been adopted to analyze text characteristics in academic research (Graesser et al., 2011). Coh-Metrix reports the average polysemy for content words in a text, and provides an incidence score for all connectives (occurrence per 1,000 words). Although the specific Coh-Metrix measures vary across versions and tools, the measures are quite similar (McNamara et al., 2014). According to the analysis results of the Coh-Metrix Web Tool (Traditional Chinese version), Text II has a larger polysemy value and a lower incidence score of connectives, which indicates a higher level of text complexity. Therefore, Task 2, which corresponds to Text II, is more complex than Task 1. The details are illustrated in Table 1. Moreover, as experts’ intuition is reasonably reliable when it comes to text complexity evaluation (Sun and Shreve, 2014), an expert panel was recruited to assess task complexity. The expert panel consisted of two translation teachers with over 5 years’ teaching experience and three professional translators with over 10 years’ translation experience. Their conclusion also indicates that Task 2 is more complex than Task 1.

**Table 1:** Task complexity and quantifiable measures of source texts.

Task	Task complexity	Text	Polysemy value of content words	Incidence score of all connectives (occurrence per 1,000 words)
1	Simple	Text I	4.692	19.231
2	Complex	Text II	5.955	12.195

#### Quality assessment metrics

The produced translation texts were evaluated by two Chinese translation teachers with over 5 years’ experience in Chinese–English translation teaching and assessment. Their assessment guidelines were adapted from Waddington’s (2001) rubric. The original rubric consists of three measures—accuracy of transfer of source text content (i.e., accuracy), quality of expression in the target language (i.e., fluency), and task completion degree. As translation quality was discussed in terms of accuracy and fluency in our study, we only considered the first two measures when developing the assessment guidelines (see Appendix B for details). Besides, for each measure, there are five levels, and each level corresponds to two possible marks; this is to comply with the marking system of 0–10, and to give raters freedom to award the mark according to whether the candidate fully meets the requirements of a particular level or falls between two levels but is closer to the upper one (Waddington, 2001).

The two raters were first invited to get familiar with the assessment guidelines and then worked together to negotiate quality assessment in the marking process. Previous studies revealed that even precise guidelines were given, cognitive bias and disagreement might still occur during the assessment process (Eickhoff, 2018; Islam et al., 2022). The negotiation approach has been widely adopted in writing assessment research and proved as an effective way to reduce raters’ bias (Trace et al., 2015). During the assessment process, the two raters analyzed product quality and reached a consensus through discussions and consultations (Yang et al., 2021b). Namely, when there was a discrepancy between their scores, the raters negotiated by providing an explanation and justification for their score assignment, in the hope of reaching a consensus score. If there was still disagreement in marking, they solved it by consulting a third person, a professional translator with over 10 years’ working experience.

### Procedure

The experiment took about two and a half hours. First, participants signed an *Informed Consent Form* approved by the university’s Ethics Committee, and completed a language background questionnaire and the Translating Self-Efficacy China scale. Then, all participants performed two translation tasks, with their task duration and translation behaviors observable on the screen recorded by screen capture software. Each of the translation tasks was followed by a subjective rating with a questionnaire adapted from NASA-Task Load Index (NASA-TLX) (Hart and Staveland, 1988), which was aimed to measure their cognitive effort invested in the preceding task. The revised NASA-TLX questionnaire, which comprises mental demand, effort, frustration, and performance subscales, is of good reliability and has been applied in some previous research to measure the amount of cognitive resources devoted to task implementation (e.g., Sun and Shreve, 2014; Sun et al., 2020; Yuan, 2022). For details, see Appendix C. To avoid sequencing effects, tasks were pseudo-randomly ordered so that participants would alternate between the two tasks. Participants were told that their translations would be assessed for external dissemination; therefore, they could review and revise their translations. This was intended to encourage participants to try their best in the experiment. This session had no time limits, and participants were allowed to access the Internet.

**Figure 2:**
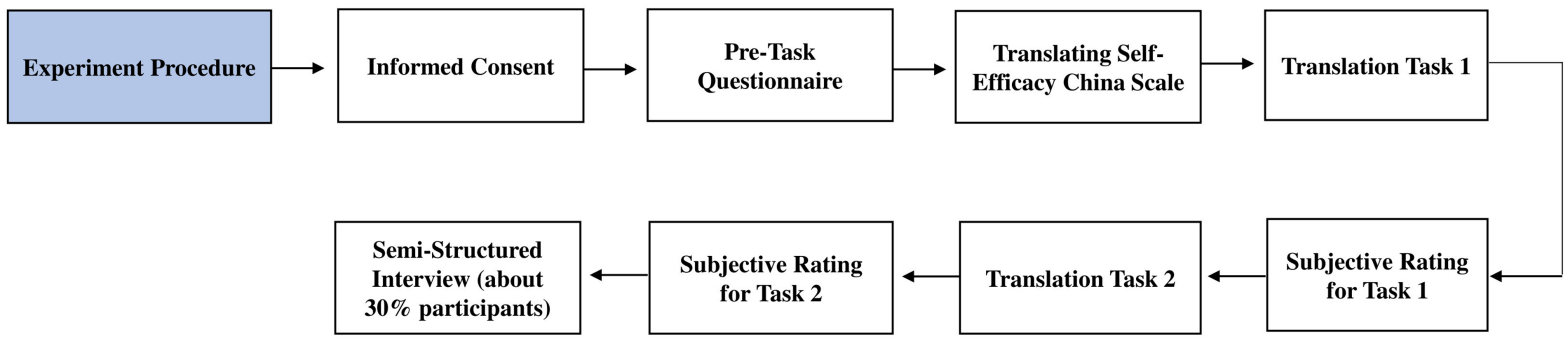
Flow chart of experiment procedure.

Upon completing the translation tasks, about 30% of the participants (*N* = 20) were randomly selected from each group to participate in a semi-structured interview, which was designed to understand students’ perceptions of task complexity and their translation performance, solution to uncertainties and ambiguities during translation, and willingness to invest cognitive effort in the process. Please refer to Figure 2 for the flow chart of the experiment procedure.

### Data quality and statistical analysis

To measure how task complexity, TSEB and their interaction influence students’ cognitive effort and translation quality, a mixed-methods approach was adopted to collect and analyze data. Specifically, subjective rating and quality evaluation were used to collect quantitative data, semi-structured interview was adopted to collect qualitative data, while screen recording was employed to collect both quantitative data (participants’ task duration) and qualitative data (participants’ translation behaviors observable on the screen). Data quality was ensured with two measures: First, EV Screen Recorder software was installed on each computer to monitor the translation process. Besides, after gathering process data, we found some outliers in the task duration and translation quality dataset. The recordings of EV Screen Recorder showed that, seven students failed to record the translation process in a complete manner or to submit their translation(s) due to technical issues with the computer. Their data were therefore excluded from the dataset. Consequently, there were 65 students in the low-TSEB group and 64 in the high-TSEB group in our data analysis.

In quantitative analysis, linear mixed-effects models (LMEMs), which can compensate for weak control of variables in naturalistic translation tasks (Saldanha and O’Brien, 2014), were employed as one of the analytical techniques to account for high variability among participants and increase the power of tests (Mellinger and Hanson, 2018). We built four LMEMs altogether. The dependent variable of the four models was (1) time-on-task, (2) self-reported cognitive effort, (3) accuracy score, and (4) fluency score, respectively. Burnham and Anderson (2004) provided rules of thumb when assessing plausible models. They believed that the best model was considered as the one with the lowest Bayesian information criterion (BIC) value. Obtained results in this study suggested that the models with interaction were better than the null ones.

For all four models, the random effects were always the participants, while the fixed effects were task complexity (simple and complex) and TSEB (low and high). As previous studies revealed a strong correlation between L2 proficiency and translation performance (e.g., Jiménez Ivars et al., 2014; Pokorn et al., 2019), the influence of L2 proficiency was controlled by adding it to the four LMEMs as a covariate. During data analysis, we first verified whether there was a significant main effect and then checked the interaction effect of task complexity and TSEB. All statistical analyses were run on IBM SPSS Statistics 26. The significance level was set at *p* = 0.05. Cohen’s *f^2^* was used to measure the effect size. The results of the four LMEMs are discussed in the following section.

## Results

### Process feature: Time-on-task

The first dependent variable in our LMEMs is time-on-task. Measured by the time from task onset to task completion, time-on-task has often been used as a measure of cognitive effort (Sweller et al., 2011). Overall, the first LMEM showed a significant main effect of task complexity (*b* = −215.625, *SE* = 38.239, *t* = −5.639, *p* < 0.001, 95% CI −291.323 ~ −139.927, *f^2^* = 0.112); but neither TSEB nor the interaction of the two independent variables proved significant (*p* > 0.05; *p* > 0.05). Table 2 shows the descriptive statistics for time-on-task in the simple and complex tasks for both groups, and the interaction between task complexity and TSEB. As indicated by task duration, the complex task engaged more cognitive effort than the simple one.

**Table 2:** Time-on-task—significant effect of task complexity.

Effect	Descriptive statistics for time-on-task in seconds				
	Factor level	Factor level	*N*	Mean	*SE*
Task complexity (TC)	Simple	/	129	1259.217	26.618
	Complex	/	129	1465.338	26.618
TSEB	Low	/	129	1376.701	31.562
	High	/	129	1347.854	33.355
TC*TSEB	Simple	Low	65	1278.393	36.825
		High	64	1240.042	38.446
TC*TSEB	Complex	Low	65	1475.008	36.825
		High	64	1455.667	38.446

### Process feature: Self-reported cognitive effort

The second variable in our LMEMs is self-reported cognitive effort, which was measured with the revised NASA-TLX questionnaire mentioned above. Regarding the measurement of cognitive effort invested in task implementation, self-rating scales were more sensitive and far less intrusive (Sweller et al., 2011). The overall results showed statistically significant effect of task complexity (*b* = −0.383, *SE* = 0.130, *t* = −2.938, *p* < 0.01, 95% CI −0.641 ~ −0.125, *f^2^* = 0.024); however, the main effect of TSEB and the interaction effect of task complexity and TSEB did not reach statistical significance (*p* > 0.05; *p* > 0.05). Table 3 summarizes the descriptive statistics for self-reported cognitive effort. According to self-ratings of cognitive effort, students put in more cognitive effort in the complex task than in the simple one.

**Table 3:** Self-reported cognitive effort—significant effect of task complexity.

Effect	Descriptive statistics for self-reported cognitive effort				
	Factor level	Factor level	*N*	Mean	*SE*
Task complexity (TC)	Simple	/	129	5.493	0.089
	Complex	/	129	5.826	0.089
TSEB	Low	/	129	5.703	0.105
	High	/	129	5.615	0.111
TC*TSEB	Simple	Low	65	5.561	0.123
		High	64	5.424	0.129
TC*TSEB	Complex	Low	65	5.846	0.123
		High	64	5.807	0.129

Table 4 provides details of an independent samples *t*-test for mental demand rating, a subscale of the revised NASA-TLX questionnaire. The table shows that the mean perceived mental demand of Task 1 and Task 2 were 5.51 and 6.03 in the low-TSEB group, and were 5.75 and 6.00 in the high-TSEB group, respectively. The two groups did not vary significantly in the perceived mental demand of Task 1 and Task 2, respectively (*t* = −0.822, *p* > 0.05; *t* = 0.108, *p* > 0.05). In addition, to assess whether the self-rated mental demand of Task 1 and Task 2 differed significantly in each group, a paired samples *t*-test was employed for both groups. The results showed that the perceived mental demand increased significantly from Task 1 to Task 2 for the low-TSEB group (*t* = −2.790, *p* < 0.01), but not for the high-TSEB group (*t* = −1.183, *p* > 0.05). In a word, the two groups had similar perceptions of task demands in Task 1 and Task 2, respectively; besides, only the low-TSEB group realized a significant increase in task demands when task complexity changed from simple to complex.

**Table 4:** Independent samples *t*-test for mental demand subscale.

Task	Group	*N*	Mean	*SD*	Independent samples *t*-test	
					*t*	Sig. (two-tailed)
1	Low-TSEB	65	5.51	1.501	−0.822	0.413
	High-TSEB	64	5.75	1.834	/	/
2	Low-TSEB	65	6.03	1.714	0.108	0.914
	High-TSEB	64	6.00	1.512	/	/

Table 5 summarizes the results of an independent samples *t*-test for performance rating, which is also a subscale of the revised NASA-TLX questionnaire and ranges from good (coded as one point) to poor (coded as 10 points). It is shown that the mean perceived quality of Task 1 and Task 2 were 5.72 and 5.63 in the low-TSEB group, and were 5.13 and 5.75 in the high-TSEB group, respectively. The two groups differed significantly in the perceived quality of Task 1 (*t* = 2.091, *p* < 0.05), but not in that of Task 2 (*t* = −0.447, *p* > 0.05). In short, the high-TSEB group was significantly more confident in their translation quality than the low-TSEB group in the simple task. But this was not true for the complex task.

**Table 5:** Independent samples *t*-test for performance subscale.

Task	Group	*N*	Mean	*SD*	Independent samples *t*-test	
					*t*	Sig. (two-tailed)
1	Low-TSEB	65	5.72	1.452	2.091	0.039
	High-TSEB	64	5.13	1.777	/	/
2	Low-TSEB	65	5.63	1.409	−0.447	0.655
	High-TSEB	64	5.75	1.613	/	/

### Product feature: Accuracy

The quality of translation products is analyzed in terms of accuracy and fluency. In this paragraph, the dependent variable discussed is the accuracy score. The overall results revealed that both fixed factors (task complexity and TSEB) significantly influenced translation accuracy (*b* = 1.625, *SE* = 0.121, *t* = 13.457, *p* < 0.001, 95% CI 1.386 ~ 1.864, *f^2^* = 0.456; *b* = −0.405, *SE* = 0.198, *t* = −2.044, *p* < 0.05, 95% CI −0.796 ~ −0.014, *f^2^* = 0.017). However, their interaction effect was not significant (*p* > 0.05). In other words, students produced a significantly less accurate translation in the complex task than in the simple one, regardless of their TSEB level. Besides, students with high TSEB significantly outperformed their counterparts with low TSEB in terms of accuracy in both tasks. The descriptive statistics for accuracy are provided in Table 6.

**Table 6:** Accuracy—significant effects of task complexity and TSEB.

Effect	Descriptive statistics for accuracy				
	Factor level	Factor level	*N*	Mean	*SE*
Task complexity (TC)	Simple	/	129	6.692	0.099
	Complex	/	129	5.134	0.099
TSEB	Low	/	129	5.677	0.122
	High	/	129	6.148	0.131
TC*TSEB	Simple	Low	65	6.423	0.136
		High	64	6.961	0.144
TC*TSEB	Complex	Low	65	4.931	0.136
		High	64	5.336	0.144

### Product feature: Fluency

The fourth LMEM was built with the fluency score as the dependent variable. Statistically significant effects of task complexity and TSEB were found on translation fluency (*b* = 1.750, *SE* = 0.099, *t* = 17.599, *p* < 0.001, 95% CI 1.553 ~ 1.947, *f^2^* = 0.391; *b* = −0.666, *SE* = 0.222, *t* = −3.003, *p* < 0.01, 95% CI −1.104 ~ −0.228, *f^2^* = 0.042). However, the interaction effect of task complexity and TSEB did not reach statistical significance (*p* > 0.05). This means that both groups produced significantly poorer fluency in the complex task than in the simple one. In addition, high-TSEB students achieved significantly greater fluency than low-TSEB students. The descriptive statistics for fluency are provided in Table 7.

**Table 7:** Fluency—significant effects of task complexity and TSEB.

Effect	Descriptive statistics for fluency				
	Factor level	Factor level	*N*	Mean	*SE*
Task complexity (TC)	Simple	/	129	5.801	0.111
	Complex	/	129	4.142	0.111
TSEB	Low	/	129	4.593	0.143
	High	/	129	5.350	0.155
TC*TSEB	Simple	Low	65	5.378	0.151
		High	64	6.225	0.162
TC*TSEB	Complex	Low	65	3.809	0.151
		High	64	4.475	0.162

## Discussion

The Results section shows complex effects of task complexity and TSEB on students’ translation process and product quality, and prove the importance of TSEB in investigating the impact of task complexity on translation performance. We found that the complex task led to significantly longer time-on-task, greater self-reported cognitive effort, lower accuracy, and poorer fluency than the simple one in both groups. Moreover, the high-TSEB group achieved significantly higher accuracy and greater fluency when compared with the low-TSEB group in both tasks. However, the interaction effect of task complexity and TSEB was not statistically significant. The findings are further discussed in the following paragraphs.

### Effects of task complexity on translation performance

#### Effect of task complexity on cognitive effort

Irrespective of their TSEB level, students put in a higher level of cognitive effort in the complex task as measured by the time-on-task and self-reported cognitive effort. Our finding corresponds to some previous findings that complex tasks engage greater cognitive effort. For example, Feng (2017) reported that L2 translation, which was cognitively more demanding than L1 translation, involved greater cognitive effort as indicated by longer task duration. Besides, Wang (2022) also concluded that translation students invested more cognitive effort in the complex task than in the simple one, which was indicated by their longer production time and longer pausing time.

The time-consuming effect resulting from task complexity may be explained by differences in participants’ strategic behaviors since cognitive load can impact mental processes (Muñoz Martín, 2014). Analysis of the screen recordings gave us some insights into students’ translation process. First, regarding cognitive resources allocated to the three phases of translation (Jakobsen, 2002), students’ time on initial orientation increased with task complexity, although they generally spent short time on initial orientation in both tasks. Participant 62 spent about 20 s on initial orientation in the simple task, compared to 105 s in the complex task. For Participant 111, her orientation time on the simple and the complex tasks were 25 and 75 s, respectively. Second, when faced with higher task complexity, students tended to improve their output by monitoring the translation process during both the drafting and the revision stages. For example, Participants 42 and 80 exhibited a higher level of product monitoring (evaluation) in the complex task than in the simple one. Finally, students had a higher frequency of pauses in the complex condition than in the simple condition, such as Participants 16 and 88. Angelone (2010) proposed that pauses or hesitations were a diagnostic sign of uncertainties in the problem-solving process, which could occur at any translation phase. Such uncertainties might cause students to doubt their comprehension of the source text, ability to work out a solution, or solution evaluation capacity.

However, it is interesting to find out that no statistically significant correlation was observed between time-on-task and self-reported cognitive effort (Pearson’s r = 0.098, *p* > 0.05). This indicates that task complexity influenced the two measures of cognitive effort in a separate manner. Ogawa (2021) also revealed that task complexity may affect translators’ task duration and subjective rating in a different way. A possible explanation is that students recruited in the current study were undergraduates and had weak problem awareness, which led to underrating of cognitive effort and in turn to insignificant correlation between the two measures. Such an idea was corroborated by data from the semi-structured interview. The interview data demonstrated that students on the whole had low problem awareness. To be specific, when asked how to assess task complexity in the semi-structured interview, 18 of the 20 high-TSEB interviewees stated that task complexity depended on the frequency of new words, and only one interviewee mentioned the number of connectives. In the low-TSEB group, 16 of the 20 interviewees responded that they evaluated task complexity based primarily on the number of new words; besides, topic familiarity, lexical polysemy, and use of connectives were, respectively, mentioned by two interviewees. Such a finding highlights the importance of recruiting students with diverse education backgrounds in future studies so as to compare their performance.

#### Effect of task complexity on product quality

A significant main effect of task complexity was observed on the accuracy and fluency scores. Higher task complexity led to poorer translation quality. Our finding lends support to Michael et al. (2011), who claimed that ambiguous words, indicative of high complexity, were translated less accurately as compared to unambiguous words. Whyatt (2019) reported that participants made more grammar mistakes in L2 translation than in the less demanding L1 translation, indicating that higher task demands led to reduced fluency. Wu (2019) also reported that higher text complexity led to greater inaccuracy and dysfluency in students’ performance.

However, the finding conflicts with Sun et al. (2020), who arrived at their conclusion when using a complex text as the source material and operationalizing task complexity as the number of simultaneous tasks. According to Cognitive Load Theory, students generally “increase cognitive effort to match increasing task demands up until they reach the limit of their mental capacities” (Chen et al., 2016: 3). As a result, with an increase in task complexity, students can adjust their level of cognitive effort to maintain the quality level achieved in the less complex task. That explains why students’ translation quality had no significant change when the condition changed from single-task to dual-task in Sun et al. (2020).

Previous research showed that the relationship between cognitive effort and performance quality was not linear: Increased effort may lead to enhanced, unchanged, or reduced quality depending on whether task complexity is low, moderate, or high (Charlton, 2002; Seufert et al., 2017). In the current study, students produced poorer translation quality in the complex task despite investment of more cognitive effort, as they, with weak problem awareness, failed to adequately increase their cognitive effort to match increasing task demands and properly tackle the translation problems.

### Effects of TSEB on translation performance

#### Effect of TSEB on cognitive effort

The low-TSEB and high-TSEB groups were similar in cognitive effort as per time-on-task and self-reported cognitive effort. Such a finding contradicts Araghian et al. (2018), who found that high self-efficacy led participants to spend less time on the translation task as highly efficacious students had greater confidence in dealing with larger translation units and reported fewer lexical and sentential problems. However, the comparison should be made with caution since the task involved in their study was translating an English text into Persian, a low-resource language (Fadaei and Faili, 2020). Translating from a high-resource language to a low-resource language poses challenges related to word ordering (Fadaei and Faili, 2020), semantic and sentence representations (Gu et al., 2018), and so on. It is different from Chinese-to-English translation implemented in the current study, as both Chinese and English are high-resource languages (Aysa et al., 2022).

According to Cognitive Load Theory, devoting greater cognitive effort is on the condition that task performers have consciously realized increased task demands and/or feel motivated to do so (Chen et al., 2016). However, first, the two groups had similar mental demand ratings (i.e., perceived task demands) in Task 1 and Task 2, respectively (see section Process feature: Self-reported cognitive effort for more details). This was corroborated by the interview data. As is mentioned in section Effect of task complexity on cognitive effort, although the low-TSEB group performed slightly better in assessing task complexity than their counterparts, students overall had weak problem awareness as they mainly referred to new words for complexity assessment. In this study, task complexity was operationalized as word polysemy value and incidence score of connectives. Ignorance of translation problems resulted in their failure to accurately assess processing demands of the complex task and in turn adequately increase cognitive effort to match increased task demands. Our finding corresponds to the finding of Jääskeläinen (1996: 67) that students “translate quickly and effortlessly” because they problematized less than semi-professionals.

Besides, according to the semi-structured interview, high-TSEB students were more willing to put in greater cognitive effort than their counterparts with low TSEB, but largely on the condition that “the task becomes more demanding.” However, as previously mentioned, the high-TSEB group failed to realize that Task 2 was significantly more demanding than Task 1 (see section Process feature: Self-reported cognitive effort). In other words, the two groups did not vary significantly in cognitive effort due possibly to similar perceptions of task demands in each task and lack of strong motivation to invest more cognitive effort in the complex task.

#### Effect of TSEB on product quality

TSEB had a significant effect on students’ translation accuracy and fluency. This finding lends support to Jiménez Ivars et al. (2014) who concluded that self-efficacy could boost translation quality. Given that TSEB was a strong predictor of translation quality but not of cognitive effort, it was possible that self-efficacy enhanced product quality through resourceful use of strategies rather than changing task duration, which echoes the findings of Hoffman and Schraw (2009). Araghian et al. (2018) also concluded that self-efficacy might influence students’ strategy use. According to Bandura (1993), it required a strong sense of efficacy to remain task oriented in the face of pressing demands and to effectively process information that contained many ambiguities and uncertainties. Therefore, when faced with a translation problem, students with high TSEB might be more resourceful in the allocation and adaptation of alternative strategies than the low-TSEB students, which in turn led to higher translation quality.

An analysis of data collected *via* the semi-structured interview underpins such an explanation. For example, a low-TSEB participant mentioned in the interview that she mainly resorted to external resources to reach a definitive solution to translation problems, while a high-TSEB participant stated that depending on the nature of the translation problem, she alternated between relying on her own knowledge and using external resources to reach a solution. Both internal and external support can help address unfamiliar terms whose equivalent expression in the target language is available on the Internet. However, it might be futile to resort solely to external resources when it comes to translation uncertainties and ambiguities arising from polysemous words or text cohesion. Resourceful strategy use by the high-TSEB group is indicative of their better allocation of cognitive resources during the translation process.

### Interaction effect of task complexity and TSEB on translation performance

No statistically significant interaction effect of task complexity and TSEB was found on students’ cognitive effort and product quality. Our finding is inconsistent with that of Rahimi and Zhang (2019), who identified an interaction effect between task complexity and self-efficacy. First, writing tasks were used in their study, which are different from translation, a complex cognitive task that comprises source text reading and target text production (Feng, 2017). Second, increased task complexity did not result in evident differences in the cognitive effort of the two groups because neither group put in significantly more effort in the complex task than in the simple one. These reasons could potentially explain the contradiction in the findings.

However, despite insignificant interaction effect on cognitive effort and product quality, the two groups displayed obvious differences in other aspects with increased task complexity. First, although the two groups did not expend significantly more cognitive effort in the complex task, the main reason behind their decision was different: The high-TSEB group did not devote more effort due to their failure in realizing a significant increase in task demands, whereas the low-TSEB group had low willingness to devote more effort. Second, the two groups had observable differences in quality perception in the simple task, but such differences diminished in the complex task (refer to section Process feature: Self-reported cognitive effort for details). This shows that high task complexity may reduce the effect of TSEB, which lends support to Judge et al. (2007), who believed that the role of self-efficacy was more evident in less complex tasks.

## Conclusion

This study examined students with high and low TSEB when they performed written translation tasks across two complexity levels. To the best of our knowledge, this study is the first to examine the effects of task complexity and TSEB on both the process and the product of written translation. The research questions raised at the beginning of the paper are addressed based on qualitative and quantitative analysis of data from screen recording, subjective rating, semi-structured interview, and quality evaluation.

First, the impact of task complexity was found on both the translation process and the end product of students. Irrespective of their TSEB level, students had longer task duration, higher self-ratings of cognitive effort, lower accuracy, and poorer fluency in the complex task than in the simple one. The evidence seems to reveal that, when faced with a higher level of cognitive load, students would put in more cognitive effort. However, unrealistically high cognitive load would reduce their translation quality. Second, high TSEB was associated with higher accuracy and greater fluency, but did not cause significant differences in time-on-task and self-reported cognitive effort. The evidence seems to indicate that highly efficacious students produced higher translation quality through more flexible allocation of cognitive effort rather than expending more cognitive effort in the translation process. That may also explain why the interaction effect of task complexity and TSEB was not significant on cognitive effort.

Examining the findings in this study together with those in previous studies, it becomes evident that the relationship between cognitive effort and task performance is not linear, depending on the level of task complexity. The finding proves the importance of quantifiable measures for categorizing task complexity. Otherwise, a task considered simple in one study might not be defined as such in another. Quantifiable measures were adopted in the present study to categorize task complexity, which can provide a reference for future translation studies to compare research results. Besides, the study also highlights the necessity of problem awareness cultivation among students since awareness of cognitive load increase is one prerequisite for students to put in more cognitive effort. With problem awareness in hand, students are in a better position to know what to look for in their performance so that their performance can be self-assessed, not just from the perspective of the end product, but also from the perspective of the translation process that contributes to its production.

The research findings can, firstly, inform translation teachers to gear task complexity to students’ developmental levels of translation competence and to pedagogical objectives. For instance, simple tasks can be assigned to help students build self-efficacy, and moderately complex tasks be assigned to facilitate their development (Graesser et al., 2011). If challenging tasks are assigned for a particular objective, scaffolds can be used to reduce the impact of task complexity. For example, Jia et al. (2019) found that neural machine translation can help students address terminology issues and reduce their cognitive effort when specialized texts, indicative of high complexity, were assigned to develop their background knowledge. Secondly, our findings also highlight potential benefits of TSEB. To help students benefit from high TSEB, teachers can draw on existing research findings on self-efficacy development, which relies on enactive mastery experience, vicarious experience, verbal persuasion, and physiological and emotional states (Bandura, 1997). Lastly, from a methodological perspective, we believe that the integrated approach adopted in this study, namely combining process and product data for translation performance research, allows us to bring to light results that might have been more difficult to identify using the onefold approach. By observing participants’ translation process, and not only their products, future studies may develop a better understanding of translation performance.

Although a mixed-methods design was adopted to collect data from several sources for triangulation purposes, this study still has some limitations. First, key-logging and eye-tracking data could be utilized to better observe students’ translation behaviors, so as to illustrate and explain their translation process more vividly. Second, there are only a limited number of source texts, single text type and language pair, and students with similar education background involved in the experiment. Third, the current study focuses on human translation. Considering the recent success of neural machine translation (Almansor and Al-Ani, 2018; Islam et al., 2021), it will contribute further to translation performance research if different task types (i.e., human translation, and post-editing of neural machine translation) are taken into account. Future studies could diversify the design of task features (e.g., task type) and select participants with different language pairs and diverse education backgrounds, so as to explore further the relationships between variables in task complexity, learner factors, and translation performance with larger samples.

## Data availability statement

The raw data supporting the conclusions of this article will be made available by the authors, without undue reservation.

## Ethics statement

The studies involving human participants were reviewed and approved by Ethics Committee of Hunan University. The participants provided their written informed consent to participate in this study.

## Author contributions

XZ and XW contributed to the conception of the study. XZ conducted the experiment and drafted the manuscript. XW and XL contributed to the revision of the manuscript. All authors contributed to the article and approved the submitted version.

## Funding

This research was funded by National Social Science Foundation of China (grant no. 22BYY015), Education Department of Hunan Province (grant no. 21C0791) and Social Science Evaluation Committee of Hunan Province (grant no. XSP22YBZ035).

## Conflict of interest

The authors declare that the research was conducted in the absence of any commercial or financial relationships that could be construed as a potential conflict of interest.

## Publisher’s note

All claims expressed in this article are solely those of the authors and do not necessarily represent those of their affiliated organizations, or those of the publisher, the editors and the reviewers. Any product that may be evaluated in this article, or claim that may be made by its manufacturer, is not guaranteed or endorsed by the publisher.
