# Supplementary material for: The impact of task complexity and translating self-efficacy belief on students’ translation performance: Evidence from process and product data
Source: Front Psychol. 2022 Nov 3;13:911850. doi: 10.3389/fpsyg.2022.911850 (PMC9671207; doi:10.3389/fpsyg.2022.911850)
Supplement: Supplementary file 1 [file Data_Sheet_1.pdf]

Supplementary Material

1 Supplementary Data

1.1 Appendix A. Source Texts

Text I:
越来越多的中国人现在的确离不开手机了。他们中的许多人，包括老年人，都使用手机应用程序（apps）保持联系并拓宽朋友圈。他们也用手机购物、查找信息，因为手机便于携带。此外，使用手机应用程序通信比传统电话便宜。然而，这种新趋势导致人们在社交时过度依赖手机。事实上，一些年轻人已经变得十分上瘾，以至于忽视了与家人和朋友面对面的交流。

Text II:
手机刷新了人与人的关系。会议室门口通常贴着一条通告：请与会者关闭手机。可是会议室里的手机铃声仍然响成一片。我们都是普通人，并没有多少重要的事情。尽管如此，我们也不会轻易关掉手机。打开手机象征我们与这个世界的联系。手机反映出我们的“社交饥渴症”。最为常见的是，一个人走着走着突然停下来，眼睛盯着手机屏幕发短信。他不在乎停在马路中央还是厕所旁边。

1.2 Appendix B. Quality Assessment Guidelines

Measure	Rating scale	Mark
**Accuracy**	Level 5:<br>Complete transfer of ST information; only minor revision needed to reach professional standard.	9, 10
	Level 4:<br>Almost complete transfer; there may be one or two insignificant inaccuracies; requires certain amount of revision to reach professional standard.	7, 8
	Level 3:<br>Transfer of the general idea(s) but with a number of lapses in accuracy; needs considerable revision to reach professional standard.	5, 6
	Level 2:<br>Transfer undermined by serious inaccuracies; thorough revision required to reach professional standard.	3, 4
	Level 1:<br>Totally inadequate transfer of ST content; the translation is not worth revising.	1, 2
**Fluency**	Level 5:<br>Almost all the translation reads like a piece originally written in English. There may be minor lexical, grammatical or spelling errors.	9, 10
	Level 4:<br>Large sections read like a piece originally written in English. There are a number of lexical, grammatical or spelling errors.	7, 8
	Level 3:<br>Certain parts read like a piece originally written in English, but others read like a translation. There are a considerable number of lexical, grammatical or spelling errors.	5, 6
	Level 2:<br>Almost the entire text reads like a translation; there are continual lexical, grammatical or spelling errors.	3, 4
	Level 1:<br>The candidate reveals a total lack of ability to express himself adequately in English.	1, 2

1.3 Appendix C. The Revised NASA Task Load Index Questionnaire (cf. Sun and Shreve, 2014)

Mental Demand How mentally demanding was the task?

0 1 2 3 4 5 6 7 8 9 10

Low High

Effort How hard did you have to work to accomplish your level of performance?

0 1 2 3 4 5 6 7 8 9 10

Low High

Frustration How insecure, discouraged, irritated, stressed, and annoyed were you?

0 1 2 3 4 5 6 7 8 9 10

Low High

Performance How successful were you in accomplishing what you were asked to do?

0 1 2 3 4 5 6 7 8 9 10

Good Poor
